# Effects of Neonatal Overfeeding on Juvenile and Adult Feeding and Energy Expenditure in the Rat

**DOI:** 10.1371/journal.pone.0052130

**Published:** 2012-12-14

**Authors:** Aneta Stefanidis, Sarah J. Spencer

**Affiliations:** 1 Department of Physiology, Faculty of Medicine, Monash University, Melbourne, Victoria, Australia; 2 School of Health Sciences and Health Innovations Research Institute (HIRi), RMIT University, Melbourne, Victoria, Australia; St. Vincent's Institute, Australia

## Abstract

Overfeeding during perinatal life leads to an overweight phenotype that persists throughout the juvenile stage and into adulthood, however, the mechanim(s) underlying this effect are poorly understood. We hypothesized that obesity due to neonatal overfeeding is maintained by changes in energy expenditure and that these changes differ between males and females. We investigated feeding, physical activity, hormonal and metabolic alterations that occur in adult rats made obese by having been nursed in small litters (SL) compared with those from control litters (CL). There were no differences in absolute food intake between the groups, and juvenile and adult SL rats ate less chow per gram body weight than the CL did in the dark (active) phase. Juvenile, but not adult SL rats did have reduced whole body energy expenditure, but there were no differences between the groups by the time they reached adulthood. Adult SL females (but not males) had reduced brown adipose tissue (BAT) temperatures compared with CL in the first half of the dark phase. Our results indicate a persistent overweight phenotype in rats overfed as neonates is not associated with hyperphagia at any stage, but is reflected in reduced energy expenditure into the juvenile phase. The reduced dark phase BAT activity in adult SL females is not sufficient to reduce total energy expenditure at this stage of life and there is an apparently compensatory effect that prevents SL and CL from continuing to diverge in weight that appears between the juvenile and adult stages.

## Introduction

The nutritional environment in early life can be crucial in influencing body weight and has important consequences for metabolism and weight regulation throughout life. As such, overfeeding during the early postnatal period can lead to increased early weight gain that persists throughout the juvenile period and into adulthood [1], [2], [3], [4]. Any predisposition to increased weight gain is a significant risk factor for persistent obesity and the variety of health complications that are associated with it, from type II diabetes to cardiovascular disease [1], [5]. How the neonatal nutritional environment alters weight regulatory mechanisms, however, is poorly understood, and it is unknown if these mechanisms are affected in the same way in males and females.

It has previously been established that both male and female rats raised in small litters (SL), where they have greater access to their mother's milk, become overweight during the suckling period. Despite post-weaning access to identical diets to those of control litters (CL), SL rats continue to display this overweight phenotype as adults [1], [2], [3], [4]. They also have some diabetogenic disturbances, such as impaired insulin-stimulated glucose transport [6]. Much attention has been focused on food intake and the hypothalamic mechanisms regulating feeding to explain how these SL rats maintain an overweight phenotype throughout life [1], [7]. However, hyperphagia has not been consistently reported in this model. Several groups have noted no differences in food intake between CL and SL adult rats [3], [8], [9] and in cases where hyperphagia has been reported, this is either short-lived [10], and/or can be attributed to the greater size of the animal [6], [10], [11], [12]. That is, when corrected for overall body weight, SL rats do not generally eat more than CL, meaning they probably do not maintain their excess body weight through excess food intake. It is likely, therefore, that the major mechanism by which the SL rats stay overweight throughout life is via changes to metabolism and energy expenditure.

In addition to increasing body weight by increasing food intake, reductions in energy expenditure can also contribute to the maintenance of an overweight phenotype [13]. For instance, there are strong indications that the ability of the interscapular brown adipose tissue (BAT) to thermoregulate and therefore increase energy expenditure may be compromised in overweight individuals. Down-regulation of BAT function has been observed in several genetic models of obesity, with ob/ob and db/db mice having reduced levels of BAT uncoupling protein (UCP) 1 [14], [15], and obesity will develop, despite the absence of hyperphagia, in transgenic mice with a specific BAT ablation [16].

There are also indications that energy balance and propensity to become overweight may be differentially regulated in males and females. Male and female humans show differences in the propensity to become obese, with women often being more likely to develop obesity than their male counterparts and to present with indices of metabolic syndrome [17], [18], [19]. Men, on the other hand, are more likely to develop visceral obesity [20], a distribution that is particularly associated with increased risk of cardiovascular disease [21], [22]. In rodents, too, females of some strains show greater susceptibility to become obese with a high fat diet than do males [23].

We therefore hypothesized that an overweight phenotype after neonatal overfeeding is maintained by reductions in energy expenditure and that these alterations differ between males and females. Here we imposed neonatal overnutrition by manipulating the size of the litter in which the rats were raised [1], [2], [3], [4], and investigated the feeding, physical activity, hormonal, and metabolic changes that occur in animals made overweight due to neonatal overfeeding, how these are altered from the juvenile to early adult periods, and how they differ between male and female rats.

## Methods

### Ethical approval and animals

Timed pregnant Wistar rats were obtained from the Animal Resources Centre, WA, Australia. They were maintained at 22°C on a 12 hr light/dark cycle (0700–1900 hr) with pelleted rat chow and water available *ad libitum*. All procedures were conducted in accordance with the National Health and Medical Research Council Australia Code of Practice for the Care of Experimental Animals and were approved by the Monash University School of Biomedical Sciences Animal Ethics Committee.

### Litter manipulation

On the day of birth (postnatal day (P) 1) all pups were removed from their dams and randomly reallocated to new dams in litters of four or 16 as previously described [3]. Care was taken that no dam received any of her own pups. Each new litter was made up of 50% males and 50% females. Excess pups were culled.

Following pup reallocation, the litters were weighed weekly as whole litter units, it having previously been determined that males and females show similar growth rates until after weaning [24]. At weaning the pups were separated into same-sex littermate pairs and left undisturbed, except for the usual animal husbandry, until experimentation. In these experiments we report data from 248 offspring from 32 litters. In each experiment we used a maximum of two rats per sex per litter. In total we used 10 CL and 22 SL litters. Such differences in N relate to the numbers of experimental animals our manipulation generates (i.e. 16 per litter from CL and 4 per litter from SL). We did not match these in order to avoid unnecessarily killing animals. However, every experimental group contained representatives from at least three litters to control for potential maternal effects.

### Fat mass measurements

At either P14 (juveniles) or P84 (adults) a cohort of rats was deeply anaesthetized with Lethabarb (sodium pentobarbitone, Virbac, Australia; approximately 150 mg/kg intraperitoneal). We then assessed whole body composition, including adiposity, bone, and lean mass, using a small animal (juveniles) dual energy X-ray absorptiometry (DEXA; Lunar PIXImus X-Ray Densitometer System, PIXImus, Fitchburg, WI, USA) or a large animal (adults) DEXA (Hologic Discovery A, Bone Densitometer, Hologic, Inc. Bedford MA, USA; juveniles n = 4–10 rats per group, adults n = 4–11 rats per group). A second cohort of rats at these ages was anaesthetized then quickly decapitated to obtain blood samples for detection of plasma leptin (see below) The fat mass in the individual depots was determined by dissection. Inguinal fat was dissected as representative of subcutaneous fat and retroperitoneal was considered representative of abdominal fat (juveniles n = 8 rats per group, adults n = 9–12 rats per group).

### Leptin measurements

Blood samples were kept on ice until the end of the experiment, when they were centrifuged and the plasma aliquots stored at −20°C until assayed. A standard leptin ELISA (Millipore, Billerica, MA) was used to assess plasma levels. Inter-assay variability  = 2.95–3.93% coefficient of variation (CV), intra-assay variability 1.88–2.49% CV, and lower limit of detection 0.2 ng/ml. Samples from all treatment groups were assayed together (n = 6 rats per group).

### Brown adipose tissue thermogenesis

At P63, 70, or 77 a separate cohort of rats was anaesthetised with isofluorane (induced at 5% and maintained at 2%) and a sterile, silicone coated temperature-sensitive telemetry probe (Datasci, St Paul, MN, USA) implanted between the lobes of the interscapular BAT [25]. A preprogrammed temperature data logger (SubCue Dataloggers; Calgary, ALB, Canada) was also implanted in the abdomen. After four days recovery from surgery [26] the signal from the telemetry devices was recorded continuously and sampled at 5 min intervals for five days. These experiments were staggered by one week as we only had the capacity to run eight rats in the telemetry setup at any one time. The groups were randomized so that two males and two females from each group were run together (n = 6 rats per group).

### Brown adipose tissue uncoupling protein 1 measurements

One week following completion of the telemetry experiments the rats were deeply anaesthetized with Lethabarb and samples of interscapular BAT were quickly removed, snap-frozen in liquid nitrogen and stored at −80°C until ready for use. Tissue was homogenized, protein extracted, and homogenate (10 µg protein per well) separated by 15% SDS polyacrylamide gel electrophoresis as previously described [25]. Proteins were then transferred to a nitrocellulose membrane and incubated for one hr in 5% bovine serum albumin (BSA) in Tris-buffered saline, containing Tween 20 (TBS-T) then overnight at 4°C in goat anti-uncoupling protein (UCP)1 antibody (1∶250, Santa Cruz Biotechnology, Santa Cruz, CA). The membrane was then washed for 30 min in TBS-T and incubated for 1 hr in anti-goat HRP (RT, 1∶10 000, Santa Cruz Biotechnology). Bound antibodies were revealed using a chemiluminescence assay (LumiGlo kit, Cell Signalling Technologies, Danvers, MA). After detection of UCP1, membranes were washed in TBS-T then incubated with a mouse anti-β actin antibody (1∶1000; Sigma, St Louis, MO) and processed as described above (secondary antibody  =  anti-mouse HRP; 1∶4000; RT: Sigma). Densitometric analysis of both the UCP1 and actin bands was conducted using ImageJ analysis software version 1.43 g (Wayne Rasband, National Institute of Mental Health, Bethesda, MD, USA) and UCP1 to actin ratios calculated to enable a semi-quantitative analysis of UCP1 levels (n = 6 rats per group).

### Indirect calorimetry

At P25-P30 or P66, 70, or 74 the rats were placed in an indirect calorimetry system (*LabMaster*; TSE-systems, Bad Homburg, Germany) for assessment of various metabolic parameters. The experiments were staggered to allow for the capacity of the system. See ‘Brown adipose tissue thermogenesis’ above. After 48 hr acclimatization, data collection began and continued for a further 48 hr. The system monitors amounts and patterns of drinking, feeding, home cage physical activity, oxygen consumption, and carbon dioxide production. From these latter two measurements we are able to derive indications of metabolic performance such as total energy expenditure and substrate utilization where respiratory exchange ratios of 1.0 represent 100% carbohydrate oxidation and 0.7 represent 100% fat oxidation (juveniles n = 12 rats per group, adults n = 17–20 rats per group).

### Data analysis

Statistical analyses for age (juvenile/adult) were performed separately. We analysed male and female data together in the same analysis for all experiments (with the exception of pre-weaning weights where rats were weighed as whole litter units) to identify whether there were differences between the sexes. Pre-weaning body weights were compared between CL and SL rats using a one-way analysis of variance (ANOVA) with repeated measures, with litter size as the between factor and time as the repeated measure. When a significant interaction was found between litter size and time, Student's unpaired t-tests were performed for each time point. Individual rat weights and nasal-anal lengths were used to calculate a rat body mass index (rBMI) using the formula weight (g)/length (cm)2 [27]. This rBMI gives an indication of the rat's relative weight, allowing us to discount the possibility of an accelerated overall growth. Adult weights were recorded at P63 before we conducted any surgeries or changed any housing conditions. Adult weights, rBMIs, fat pad to body weight ratios, DEXA results, leptin concentrations, and UCP1 to actin ratios were compared between CL and SL and males and females using two way analyses of variance (ANOVAs) followed, where appropriate by Student Neumann-Keuls *post hoc* tests.

Food intake, water intake, energy expenditure, physical activity, oxygen consumption and carbon dioxide production were recorded every 30 min, the two latter measurements allowing us to calculate the respiratory exchange ratio (VCO_2_/VO_2_). Cumulative data for the mean 12 hr light and dark periods were compared separately using linear regression analyses. Recent discussion in the field has detailed concern that expressing absolute energy expenditure per animal fails to account for differences in overall size and correcting for total or lean mass fails to allow that metabolic rate is not linearly proportional to body weight [28]. For these reasons we used regression analysis controlling for the influence of total body weight to compare energy expenditure. As we found no influence of body weight on the groups, we have also represented the data in a histogram of mean absolute energy expenditures for comparison purposes.

Temperature data were used to derive a temperature index (°C x hr) for each 6 hr period of the light/dark cycle. Thus, the temperatures at each 5 min interval were averaged over the first 30 min to calculate a baseline for each 6 hr period. The mean change from this baseline was then calculated per hour for each animal and these data summed as appropriate to produce an ‘area under the curve’ value (temperature index) for both the core and BAT. Temperature indices were then compared for each 6 hr period using ANOVAs with Student Neumann-Keuls *post hoc* tests.

All data were assessed for homogeneity of variance and transformations applied where appropriate. Data presented as ratios were not transformed. Data are presented as the mean ± standard error of the mean (SEM). Statistical significance was assumed when *P*<0.05.

## Results

### Neonatal overfeeding affects body weight in the immediate and long-term

**Figure 1:**
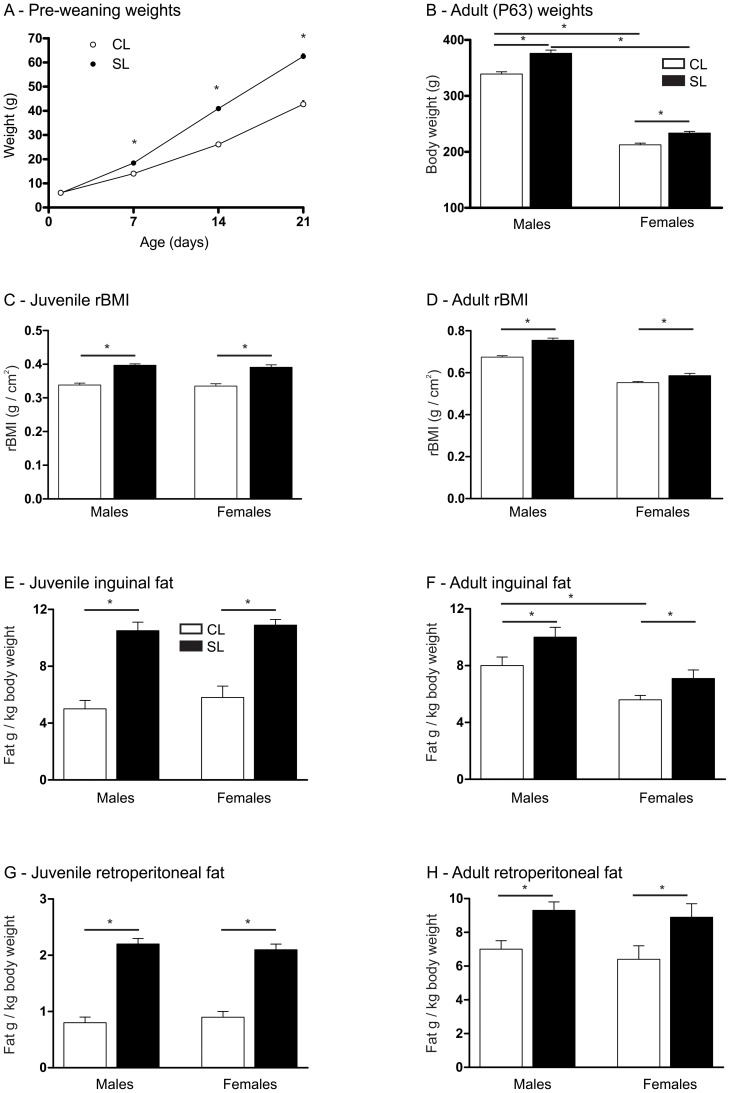
Effects of neonatal overfeeding on body weight and fat mass in the immediate and long-term. A) Pre-weaning total body weights of rats raised in control (CL) and small (SL) litters. Rats were weighed in whole litter units and weights corrected for the number of pups in the litter. n = 10 CL and 22 SL litters per group. B) Adult (postnatal day [P] 63) male and female total body weights. N = 14–28 rats per group. C) Juvenile (P21) rat body mass indices (rBMI). N = 9–13 rats per group. D) Adult (P63) rBMI. E) Juvenile left inguinal fat pad mass expressed as a ratio to total body weight. F) Adult left inguinal fat pad mass. G) Juvenile retroperitoneal fat pad mass. H) Adult retroperitoneal fat pad mass. N = 8–12 rats per group. Data are mean + SEM. * *P*<0.05.

As we [3], [4] and others [1], [2] have previously seen with this model, neonatal overfeeding led to a significantly increased body weight compared with controls and this was maintained into adulthood (Fig. 1A,B). Analysis of the rats' pre-weaning body weights revealed a significant interaction between litter size and day (*P*<0.001) with SL (n = 22 litters per group) significantly bigger than CL (n = 10 litters per group) on days 7, 14, and 21 (*P*<0.001 each), but not day one. This effect was still present in adulthood with P63 SL rats weighing significantly more than CL rats and males, as expected, weighing more than their female counterparts (significant effect of litter size: F_(3,78)_ = 41.2, *P*<0.0001, and sex F_(3,78)_ = 883.4, *P*<0.0001. Males: n = 22–28. Females: n = 14–18).

SL rats also had significantly greater rat body mass indices (rBMIs) as juveniles (Fig. 1C. Significant effect of litter size: F_(3,42)_ = 99.9, *P*<0.0001. Males: n = 12–13. Females: n = 9–12) and as adults (Fig. 1D. Significant litter size x sex interaction: F_(3,39)_ = 8.4, *P* = 0.006. Males: n = 10–12. Females: n = 9–12).

### Body weight changes with neonatal overfeeding are, at least partly, due to increased fat mass

Neonatal overfeeding elevates body weight at least partly by increasing fat mass. Thus, neonatal overfeeding resulted in a significant increase in both inguinal and retroperitoneal fat pads in absolute terms (data not shown) and when corrected for body weight (Fig. 1E–H). This increased fat mass was evident in the juvenile period and was maintained through to adulthood. Thus, juveniles had inguinal fat pads that were 1.9 (females) to 2.1 (males) times larger in the SL rats than CL (Fig. 1E. Significant effect of litter size: F_(3,28)_ = 72.6, *P*<0.0001. N = 8 for all groups). Adult SL rats had inguinal fat pads 1.3 (males and females) times larger than those of the CL rats (Fig. 1F. Significant effect of litter size: F_(3,39)_ = 9.4, *P* = 0.004, and sex: F_(3,39)_ = 22.4, *P*<0.0001. Male: n = 10–12. Female: n = 9–12).

Visceral fat is thought to be particularly associated with an increased risk for cardiovascular disease [21], [22], thus it is noteworthy that retroperitoneal fat was also affected by neonatal overfeeding. Juveniles had retroperitoneal fat pads that were 2.3 (females) to 2.8 (males) times larger in the SL rats than CL (Fig. 1G. Significant effect of litter size: F_(3,28)_ = 165.7, *P*<0.0001). This profile was maintained into adulthood with adult SL rats having retroperitoneal fat pads 1.3 (males and females) times larger than those of the CL (Fig. 1H. Significant effect of litter size: F_(3,39)_ = 13.3, *P* = 0.001).

Body composition analysis by DEXA also revealed a significantly greater percentage total fat in the SL groups at both the juvenile and adult [26] stages (Table 1. Juveniles, significant effect of litter size: F_(3,23)_ = 73.0, *P*<0.0001. Male: n = 4–9. Female: n = 4–10. Adults, significant effect of litter size: F_(3,26)_ = 12.6, *P* = 0.001, and sex: F_(3,26)_ = 19.3, *P*<0.0001. Male: n = 4–11. Female: n = 6–9). In addition, neonatal overnutrition was associated with a greater total lean mass in both male and female juveniles, and in adult males. Adult female SL rats had a similar lean mass to their CL counterparts (Table 1. Juveniles, significant effect of litter size: F_(3,23)_ = 20.5, *P*<0.0001. Adults, significant interaction between litter size and sex: F_(3,26)_ = 20.3, *P*<0.0001).

**Table 1:** Changes in body composition after neonatal overfeeding in juvenile and adult rats.

Juveniles				
	Male		Female	
	CL	SL	CL	SL
Bone mass density (mg/cm2)	30.4±1.0	33.5±0.8	30.8±0.6	33.5±1.2
Lean mass (g)	21.8±1.0	28.6±1.2*	20.8±1.4	27.3±1.2*
Lean mass (% of total mass)	88.2±0.5	79.1±1.1*	87.7±0.7	79.5±1.9*
Fat mass (% of total mass)	11.9±0.5	20.8±1.2*	12.2±0.7	20.6±2.0*

Adults				
	Male		Female	
	CL	SL	CL	SL
Bone mass density (mg/cm2)	179.6±4.0	191.0±2.7	163.3±2.4	169.5±1.6#
Lean mass (g)	338.5±5.1	406.9±12.9*	208.5±5.2#	215.4±5.8#
Lean mass (% of total mass)	78.2±0.9	73.8±1.9*	83.1±1.0#	78.8±1.6*#
Fat mass (% of total mass)	19.0±0.9	23.6±1.9*	13.7±0.9#	18.0±1.6*#

N = 4–11 rats per group. Data are mean ± SEM. * Significantly different from CL counterpart with one way ANOVA followed by Student Neuman Keuls *post hoc* test. # Significantly different from male counterpart.

### Neonatal overfeeding is associated with elevated juvenile and adult plasma leptin levels

**Figure 2:**
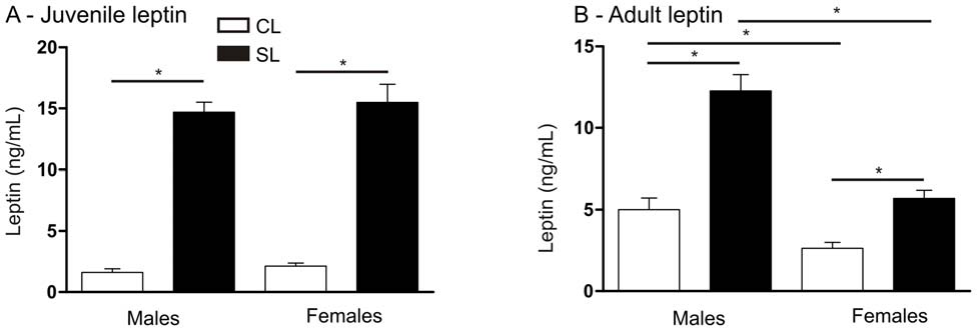
Effects of neonatal overfeeding on plasma leptin concentrations. A) Juvenile plasma leptin. B) Adult plasma leptin. Rats raised in control litters  =  CL, small litters  =  SL. N = 6 rats per group. Data are mean + SEM. * *P*<0.05.

In accordance with the elevated fat mass in the SL rats, we also saw significantly elevated plasma leptin concentrations in male and female SL compared with CL. This was the case in both juveniles and adults (Fig. 2. Juveniles, significant effect of litter size: F_(3,28)_ = 233.3, *P*<0.001. N = 8 per group. Adults, significant litter size x sex interaction: F_(3,40)_ = 9.3, *P = *0.004. N = 11 per group).

### Neonatal overfeeding does not cause post-weaning hyperphagia

**Figure 3:**
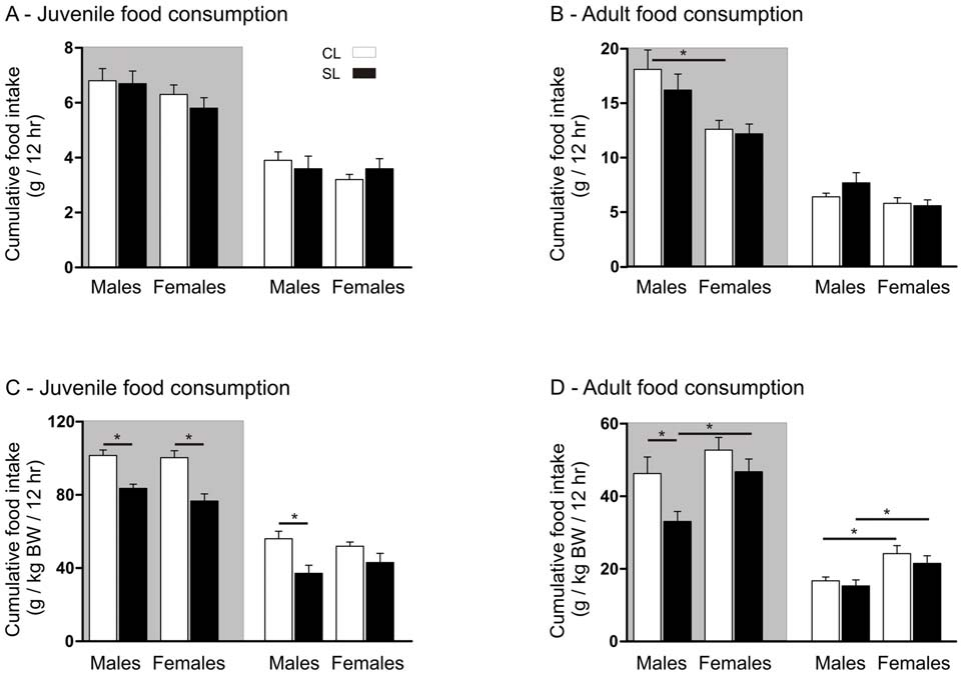
Effects of neonatal overfeeding on food intake. A) Juvenile 12 hr food consumption. B) Adult 12 hr food consumption. C) Juvenile 12 hr food consumption corrected for body weight. D) Adult 12 hr food consumption corrected for body weight. Rats raised in control litters  =  CL, small litters  =  SL. N = 12 rats per group for juveniles and 17–20 rats per group for adults. Data are mean + SEM. * *P*<0.05. Shaded area  =  dark period, clear area  =  light period.

To determine the proximal causes of the elevated fat mass and total body weight in neonatally overfed animals, we measured food intake and indices of metabolic function in the two groups. Juvenile rats from the CL and SL groups consumed similar absolute amounts of food in both the dark and light periods (Fig. 3A. N = 12 per group). When food consumption was corrected for body weight, juvenile rats from SL, despite their obese phenotype, actually ate less chow per gram of total body weight than did the CL rats in the dark phase, and the male SL rats continued this profile in the light. (Fig. 3C. Dark, significant effect of litter size: F_(3,42)_ = 30.0, *P*<0.0001. Light, significant effect of litter size: F_(3,42)_ = 11.4, *P* = 0.002).

The rats showed a similar feeding pattern as adults (n = 17–20 per group) with no differences in absolute food consumption, except for between the sexes in the dark phase (Fig. 3B. Dark, significant effect of sex: F_(3,69)_ = 12.4, *P* = 0.001), and a dark-phase hypophagia, at least in the males, when corrected for size (Fig. 2B, D. Dark, significant effect of litter size: F_(3,69)_ = 6.6, *P* = 0.012. Light, significant effect of sex: F_(3,69)_ = 14.1, *P*<0.0001). There were no significant differences in water consumption between the groups at any stage (data not shown).

### Neonatal overfeeding does not lead to reduced physical activity

Patterns of locomotor activity were recorded to attempt to account for the persistent overweight phenotype in our SL rats. However, no differences between the groups were seen in locomotor activity at either the juvenile or adult stages. Thus, juvenile CL and SL males displayed cumulative activity counts of 16774±750 and 18302±987 respectively in the dark phase, and 9863±647 and 11217±686 in the light phase. Juvenile CL and SL females had dark phase activity counts of 15663±703 and 15885±695 respectively, and light phase activity counts of 11715±1405 and 11043±627 (n = 12 per group). In adulthood CL and SL male activity counts were 18976±1396 and 17828±900 respectively in the dark phase and 6463±289 and 7069±283 in the light phase, while adult CL and SL females had similar activity counts of dark phase 21586±1234 and 19133±1116 and light phase 9052±399 and 8146±537 (n = 17–20 per group).

### Neonatal overfeeding causes a reduction in basal brown adipose tissue heat production in females, in the first half of the dark phase, but not in males

**Figure 4:**
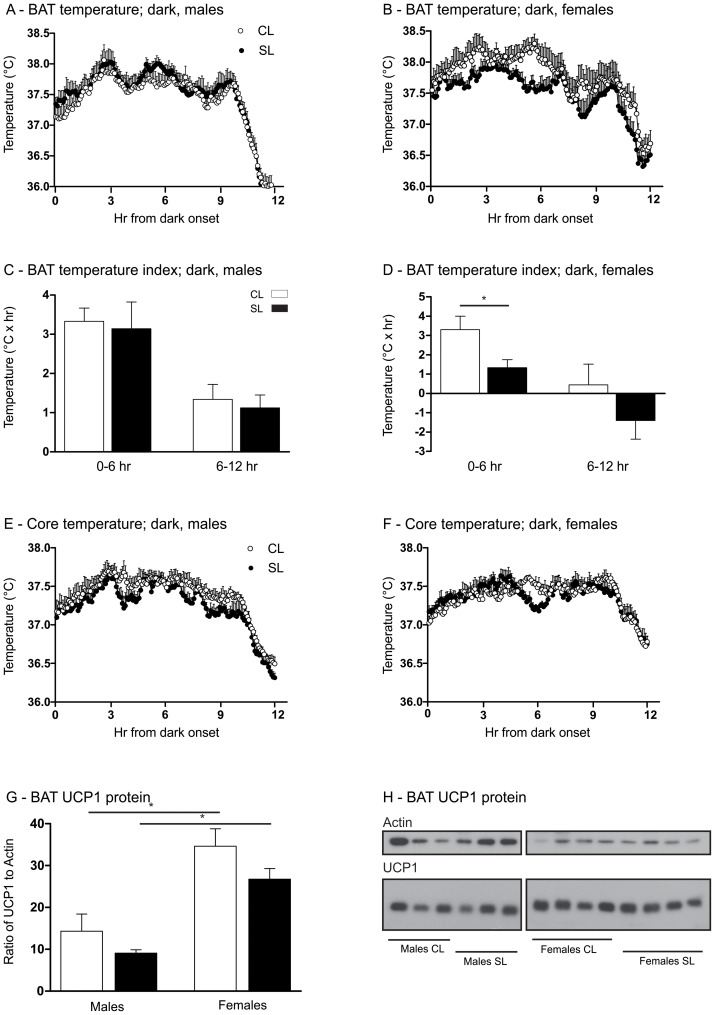
Effects of neonatal overfeeding on basal brown adipose tissue (BAT) thermogenesis. A) Male BAT temperature, dark period. B) Female BAT temperature, dark period. C) Male BAT temperature index, dark period. D) Female BAT temperature index, dark period. E) Male core temperature, dark period. F) Female core temperature, dark period. G) Densitometric analysis of BAT UCP1. H) Sample Western blot of BAT UCP1 and actin. Rats raised in control litters  =  CL, small litters  =  SL. N = 6 rats per group. Data are mean + SEM. * *P*<0.05.

In the absence of changes to eating patterns and activity, we examined heat production from BAT, as an index of BAT thermogenesis, to partially account for how neonatally overfed animals may stay overweight into adulthood. Brown adipose tissue temperature, reflective of local thermogenic activity, was significantly reduced in female, but not male, SL rats during the first half of the dark cycle in comparison to CL (Fig. 4A–C. Significant interaction between litter size and sex: F_(3,20)_ = 4.8, *P* = 0.046. N = 6 per group). Thus, the temperature index for the first half of the dark cycle for CL females was 3.31±0.69 and for SL females was 1.33±0.42°Cxhr. We saw no differences in light cycle BAT temperatures between the groups (data not shown). Basal core temperatures were not significantly different between the CL and SL groups for either males or females when the entire 6 hr period was taken into account (Fig. 4E, F). There was a significant effect of litter size (F_(3,20)_ = 4.9, *P* = 0.039) and sex (F_(3,20)_ = 40.8, *P*<0.0001) between the groups in BAT levels of UCP1 protein (Fig. 4G,H. N = 6 per group) but individual group differences within the sexes were not significant with the SNK post hoc test. (Fig. 4G, H. N = 6 per group).

### Neonatal overfeeding reduces whole body energy expenditure in juveniles, but not adults

Given our findings that SL rats were not hyperphagic, and considering the reduced BAT thermogenesis we observed in females, we expected the overweight phenotype in the SL group would be reflected in reduced resting whole body energy expenditure. This proved to be the case in the juvenile but not adult stage.

**Figure 5:**
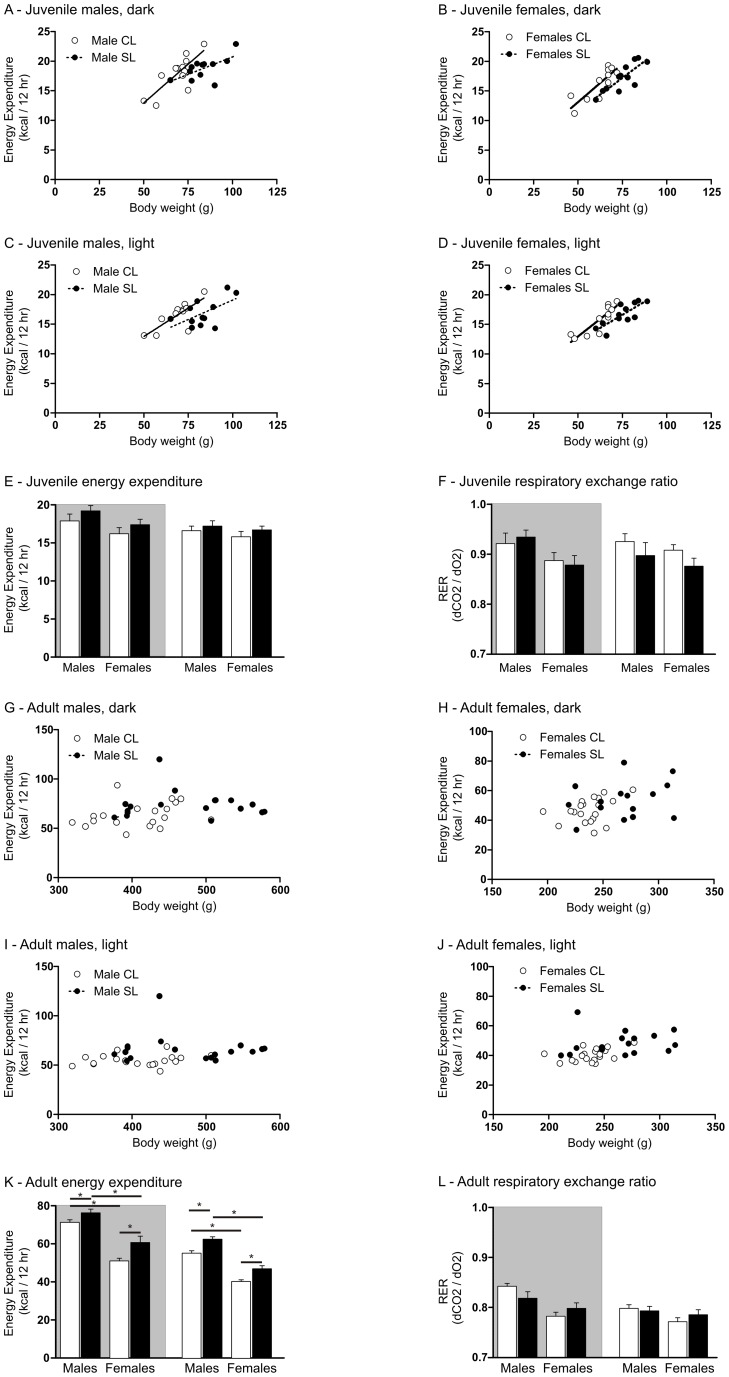
Effects of neonatal overfeeding on energy expenditure. Scatterplots show lines of best fit for energy expenditure as a function of group and body weight for A) Juvenile males, dark phase. B) Juvenile females, dark phase. C) Juvenile males, light phase. D) Juvenile females, light phase. E) Mean juvenile 12 hr energy expenditure. F) Mean juvenile respiratory exchange ratio. G) Adult males, dark phase. H) Adult females, dark phase. I) Adult males, light phase. J) Adult females, light phase. K) Mean adult 12 hr energy expenditure. L) Mean adult respiratory exchange ratio. Rats raised in control litters  =  CL, small litters  =  SL. N = 12 rats per group for juveniles and 17–20 rats per group for adults. Data are mean + SEM. * *P*<0.05. Shaded area  =  dark period, clear area  =  light period.

In the juvenile phase, energy expenditure was increased as body weight increased in both male and female rats (the linear regression line of best fit slopes were significantly different from zero in all cases; *P*<0.05; Fig. 5A–F; n = 12 per group), but this relationship was not different between the groups (no differences in slopes between groups). Thus, total energy expenditure increases as total weight increases irrespective of litter size background. Examining the elevation of the regression line allows us to interpret whether this increase in energy expenditure with increased body weight occurs to the same degree in both groups. We see the body weight-independent component of energy expenditure was significantly higher in CL males in the light phase and in SL females in both the dark and light phases compared with SL (significant differences in elevation in males, light: F_(1, 21)_ = 4.9, *P* = 0.038; females, dark: F_(1,20)_ = 8.1, *P*<0.01; females, light F_(1,20)_ = 8.5 *P* = 0.008). That is, there is less increase in energy expenditure for every gram increase in body weight in the SL than there is in the CL groups. When energy expenditure was expressed as an uncorrected mean, there was a significant effect of sex but not litter size (F_(3,43)_ = 6.0, *P* = 0.019), and this only in the dark phase. Respiratory exchange ratios were not different between the groups (Fig. 5F).

By adulthood, the influence of body weight on energy expenditure had dissipated (the linear regression line of best fit slopes were no longer significantly different from zero) and there were also no differences between the groups, i.e. indicating energy expenditure did not change as a function of body weight, and energy expenditure as a function of body weight was not different between groups (Fig. 5G–L; n = 17–20 per group). In contrast to our findings for the juveniles, the body weight-independent component of energy expenditure (how much energy expenditure increases per gram body weight increase) was significantly higher in adult SL animals (at least in the light phase) compared with CL(significant differences in elevation in males, light: F_(1, 35)_ = 8.6, *P* = 0.007; females, light F_(1,33)_ = 8.8 *P* = 0.006).

When expressed as whole body energy expenditure (i.e. per animal) it was evident that SL rats actually used more energy in both the light and dark phases than their CL counterparts (dark, significant effect of litter size: F_(3,69)_ = 77.7, *P*<0.0001, and sex: F_(3,69)_ = 12.9, *P*<0.0001. Light, significant effect of litter size: F_(3,69)_ = 31.2, *P*<0.0001, and sex: F_(3,69)_ = 148.2, *P*<0.0001. N = 17–20 per group). Respiratory exchange ratios were again not different between the groups (Fig. 5L).

## Discussion

The neonatal nutritional environment certainly plays an important role in programming long-term mechanisms that regulate feeding and metabolism. For instance, it has long been established that rodents suckled in an environment of overnutrition, as they are in small litters, have accelerated weight gain and maintain higher body weights into adulthood [1], [2], [3], [4]. We reaffirm these findings in the present study with our model of litter size manipulation. Our analysis of individual fat pad weights and DEXA images shows male and female juveniles suckled in small litters have greater lean and percentage fat mass than their control-litter counterparts. These findings are suggestive of an accelerated overall growth (increased lean mass) that culminates in an overweight or obese phenotype (increased percentage fat mass). This phenotype is maintained into adulthood, with males from small litters continuing to display greater lean and percentage fat mass and females having greater fat mass. So, how do neonatally overfed animals maintain a higher body weight throughout life?

In our hands, at least, they do not do this by overeating, as has been previously suggested [6], [10], [11], [12]. Indeed, juveniles of both sexes and adult males actually ate less chow per gram body weight if they had been overfed in the first three weeks of life, and total food intakes were not different.

Our data do indicate that rats raised in small litters have reduced whole body energy expenditure compared with CL, until at least P25–30. For a given body weight, the juveniles raised in small litters used less energy in the indirect calorimetry assessment. In the absence of hyperphagia, it is likely this reduced energy expenditure is a major factor contributing to these rats maintaining a greater size, weight, and fat mass.

Surprisingly, adult rats raised in small litters did not have a comparable reduction in energy expenditure compared with controls. If anything, their resting energy expenditure was elevated. It thus appears that, at some point between P25–30 and P70, metabolism in our SL rats switches to a comparatively higher level of resting energy expenditure that may partially compensate for the larger weight and fat mass. In combination with a conservative food intake, similar to that of controls, the effect on metabolism should be to eventually restore size and fat mass to control levels. Indeed, there is some suggestion this may be occurring in our model. The whole body effects of being suckled in a small litter appear to be more apparent at the juvenile stage: these rats have twice as much inguinal and retroperitoneal fat as juveniles than controls compared with 1.3 times as much when they are adults; they have seven to nine times as much leptin as juveniles compared with twice as much as adults; and they have eight to nine percent more total fat as juveniles compared with four to five percent more as adults, all indicating that the groups do not continue to diverge.

Despite this evidence that adult rats that were overfed as neonates may be able to compensate for their early accelerated weight gain by enhancing energy expenditure later on, it appears this ‘compensation’ is insufficient to completely reverse the overweight phenotype by P70. In females this is possibly partly due to dysregulated BAT thermogenesis during the first half of the dark phase attenuating any potential compensatory increase in energy expenditure in this phase. Brown adipose tissue is responsible for dissipating energy as heat primarily via a UCP1-mediated uncoupling of oxidative phosphorylation [13]. Impairments in BAT function can therefore result in increased energy storage. In this regard, BAT dysfunction is certainly a consistent factor across many models of obesity. Ablation of BAT will result in obesity even in the absence of hyperphagia [16], and various genetic models of obesity, such as the ob/ob and db/db mice, are also found to have reduced levels of BAT thermogenesis [14], [15].

There is some evidence from previous studies that BAT functions differently in males and females. Basal BAT thermogenesis is higher in *ad libitum* fed females than in males in a thermoneutral environment [29], [30]. Males and females also have differences in their BAT thermogenic capacity under cold and calorie restricted conditions [31], and overfeeding with a high fat diet has been shown to affect male and female BAT thermogenesis differently with female rats being unable to up-regulate BAT thermogenesis in response to a cafeteria diet, an effect associated with greater gain in weight [23].

Our present data show neonatal overnutrition in female rats induces a reduction in BAT thermogenesis in the first half of the dark phase in ambient temperatures of 22°C, as indicated by reduced heat production from this tissue. That this is not associated with a reduction in whole body energy expenditure may be a reflection of the many contributors within the body to the indirect calorimetry measurements, including potential compensatory mechanisms from other arenas that serve to elevate total energy expenditure.

In this study, male rats from small litters did not have reduced BAT thermogenesis. Furthermore, SL rats have increased lean mass as well as fat mass, which, given lean mass is usually associated with an increased metabolic rate relative to fat mass [32], one would expect to be reflected in increased total energy expenditure. Despite these factors, the overweight phenotype did not appear to be exacerbated in SL males compared with SL females; the sexes showing similar increases in fat mass and leptin. The reasons behind these discrepancies are unknown, but may reflect the myriad factors that contribute to total energy expenditure and final body composition.

In this study we provide the first evidence that rats made overweight due to overfeeding in the first three weeks of life have accelerated weight gain that is associated with reduced energy expenditure until at least P30. By P70, the neonatally overfed rats no longer use less energy than CL. If anything, SL energy expenditure is greater in adulthood, despite the females having less heat production from BAT in the first half of the dark phase. That the neonatally overfed rats are still significantly heavier at this time, and are still compromised in other aspects of their physiology, such as ability to process an immune challenge or stress, illustrates these potentially compensatory mechanisms are only partially successful [3], [26]. The mechanisms by which these metabolic pathways are altered as the animal ages are unknown and remain to be determined. However, these data may mean that the immediate postnatal period is not as crucial as first thought in establishing metabolism and body weight. Undoubtedly we are able to permanently influence metabolism and feeding during these periods in ways we are not at other times of life. However, our data imply the damage is reversible as long as moderate feeding patterns are maintained.
